# Vitamin D Receptor and Vitamin D Binding Protein Gene Polymorphisms Are Associated with Renal Allograft Outcome

**DOI:** 10.3390/nu13041101

**Published:** 2021-03-27

**Authors:** Sepideh Zununi Vahed, Elham Ahmadian, Peyman Foroughi, Soroush Mostafavi, Henning Madry, Mohammadreza Ardalan, Magali Cucchiarini

**Affiliations:** 1 Kidney Research Center, Tabriz University of Medical Sciences, Tabriz 5166615731, Iran; sepide.zununi@gmail.com (S.Z.V.); ahmadian.elham@yahoo.com (E.A.); payman.foroughi@gmail.com (P.F.); so.mostafavi@gmail.com (S.M.); 2 Center of Experimental Orthopaedics, Saarland University Medical Center, D-66421 Homburg, Germany; henning.madry@uks.eu

**Keywords:** kidney transplant, vitamin D, polymorphism, allograft rejection, viral infection, vitamin D binding protein, vitamin D receptor

## Abstract

Vitamin D deficiency has adverse effects on renal allograft outcomes, and polymorphisms of genes encoding vitamin D-binding protein (VDBP) and vitamin D receptor (VDR) are defined to play a role in these conditions. The goal of the current investigation was to evaluate the connection between those polymorphisms with acute rejection, viral infection history, and recipients’ vitamin D status. In this study, 115 kidney transplant recipients and 100 healthy individuals were included. VDR polymorphisms including *Fok*I (rs2228570), *Apal* (rs7975232), *BsmI* (rs1544410), as well as VDBP (rs7040) polymorphisms were studied using high resolution melting (PCR-HRM) analysis among the studied groups. The frequency of G allele in *Apal* rs7975232 polymorphism in the kidney transplant recipients was 0.63 times lower than healthy individuals (*p* = 0.026). Further, the G allele frequency in VDBP rs7040 polymorphism was significantly lower in patients with allograft rejection (*p* = 0.002). Considering the incidence of viral infection, significant differences were identified between the frequencies of VDR *Fok*I (OR = 2.035; 95% CI 1.06–2.89, *p* = 0.030) and VDBP rs7040 (OR = 0.40; 95% CI 0.24–0.67, *p* < 0.001) T alleles in the studied groups. Moreover, the VDBP rs7040 GG genotype distribution was low in the recipients with a history of viral infection (*p* = 0.004). VDR (*Fok*I) and VDBP (rs7040) alleles and their genotype distribution are significantly associated with allograft outcomes including allograft rejection and viral infection in the studied population.

## 1. Introduction

Kidney transplantation is associated with better survival, increased life quality, and reduced costs in comparison with dialysis [1]. However, acute graft rejection happens in around 20–40% of the recipients, and chronic rejection is still the main causes of diminished graft functionality [2,3]. Several studies have focused on the development of novel strategies to reduce transplantation rejection.

Vitamin D is a secosteroid hormone, whose metabolizing enzymes and its intracellular receptor (VDR) generate the active hormone and mediate its effect on the skeletal metabolism, tumor-inducing cascades, and immune responses [4]. Vitamin D regulates innate and adaptive immune systems and chemokine receptors [5], influencing monocytes differentiation, lymphocyte proliferation, and cytokine production [6]. Sufficient levels of vitamin D stimulate the innate immune system through the toll-like receptors (TLRs) in the immune cells. The stimulation of TLRs in the immune cell stimulates the generation of anti-microbial peptides (AMPs) such as reactive oxygen species (ROS), and cathelicidin that further kill the intracellular microorganism. Moreover, vitamin D mediates immunologic tolerance [7] as it prohibits the maturation of dendritic cells, and thus prevents the further activation of immune cells [8]. These cellular effects of vitamin D are mediated by its receptor. The activation of VDR by 1,25-dihydroxyvitamin D_3_ [1,25(OH)_2_D3], as a nuclear transcription factor, regulates the expression of more than one thousand genes in different tissues [9].

Vitamin D and all its metabolites are transported by a multifunctional vitamin D binding protein (VDBP). With a high affinity, DBP binds to 1,25(OH)_2_D_3_ and 25-hydroxyvitamin D [25OHD] that generate a circulating 25OHD pool, inhibiting rapid deficiency of vitamin D. Moreover, VDBP regulates the access of the metabolites of vitamin D to tissues and cells [10,11].

Vitamin D has also a potential effect on kidney allografts maintenance [12]. Vitamin D may decline the dose of immunosuppressive drugs (especially calcineurin inhibitors), resulting in a reduction of their nephrotoxicity and infectious complications. Moreover, according to the assisting knowledge, vitamin D can plummet the development of chronic allograft injury [13]. In this regard, deficiency of vitamin D is concomitant with adverse outcomes of transplanted allograft including reduced glomerular filtration rate (GFR) and increased risk of graft rejection and infections [14,15].

The role of genetic alterations such as polymorphisms and mutations in VDR and VDBP have also been postulated to modulate immune response after allograft transplantation [16,17,18]. However, the current data are limited and require further investigations. Therefore, the present experiment was intended to evaluate the connection between VDR and VDBP gene polymorphisms with kidney allograft function and outcomes including viral infection and allograft rejection.

## 2. Materials and Methods

### 2.1. Study Subjects

This retrospective study was conducted on the kidney transplant recipients of Imam Reza Hospital, Tabriz, Iran. The inclusion criteria were living non-related donors, having undergone kidney transplantation at least three years earlier, and both males and females gender between 15 to 60 years old. The exclusion criteria were included age ranges beyond those inclusion limits, kidney allograft malfunction related to urologic problems such as obstruction and kidney stone, a history of kidney transplantation (second or third transplantation), and kidney recipients suffering from kidney failure due to systemic or organ-specific reasons (sepsis, pneumonia, myocardial infarction, trauma, etc.). Level of vitamin D, clinical information, and medicine were recorded. Moreover, evidence on allograft rejection and a prior history of cytomegalovirus (CMV) and BK polyomavirusa (BK) infections were recorded as allograft outcomes. GFR was calculated by the formula of modification of diet in the renal disease (MDRD) GFR = 175 × (serum creatinine (mg/dL))^−1.154^ × (age in years)^−0.203^ × (0.742 if female)) [19]. Overall, the participants were categorized in four groups, based on third year allograft function defined by their GFR as the followings; acceptable allograft function was defined as (GFR > 45), moderate to severe graft dysfunction was defined as GFR ranged between (45 < GFR > 10), and those with poor allograft function had a dropped GFR below 10 mL/min/1.73 m^2^. One hundred healthy individuals were also employed as the control group and compared with the case group. The Ethics Committee of Tabriz University of Medical Sciences, Tabriz, Iran approved this work (IR.TBZMED.REC.1397.155). Subjects agreed to participate in this study by signing written informed consent.

### 2.2. Genetic Study

For running a genetic check from the individuals under study, 2^CC^ blood samples were collected and kept frozen in CBC pipes in −20 °C till DNA extraction. Genomic DNAs were extracted using a manual protocol [20]. The study of VDR polymorphisms including *Fok*I C>T (rs2228570), *Apal* T>C (rs7975232), *Bsm*I G>A (rs1544410) as well as VDBP (rs7040) polymorphisms were performed using high resolution melting (PCR-HRM) analysis. The primer sequences are listed in Table 1. The PCR cycling condition was as follows: a preliminary denaturation at 94 °C for 10 min, followed by 94 °C for 10 s, 59 °C for 30 s, and 72 °C for 20 s for 40 cycles. In order to check for the accuracy of the sample size, some samples were sent to Macrogen Company in Korea for sequencing.

### 2.3. Statistical Analysis

The data were tested for normality. For parametric factors, differences between the groups were tested via the Student t-test and expressed as mean and standard deviation (SD). For non-parametric variables, differences between the groups were tested via the Mann–Whitney U test and expressed as median (Interquartile Range). The genotype distribution of polymorphisms was tested by the Chi-square test. The correlation of these polymorphisms with graft survival and function were evaluated using Pearson coefficient correlation and logistic regression analysis. Data analysis was done using SPSS Version 22 (Chicago). *p*-value < 0.05 was considered statistically significant.

## 3. Results

**Figure 1:**
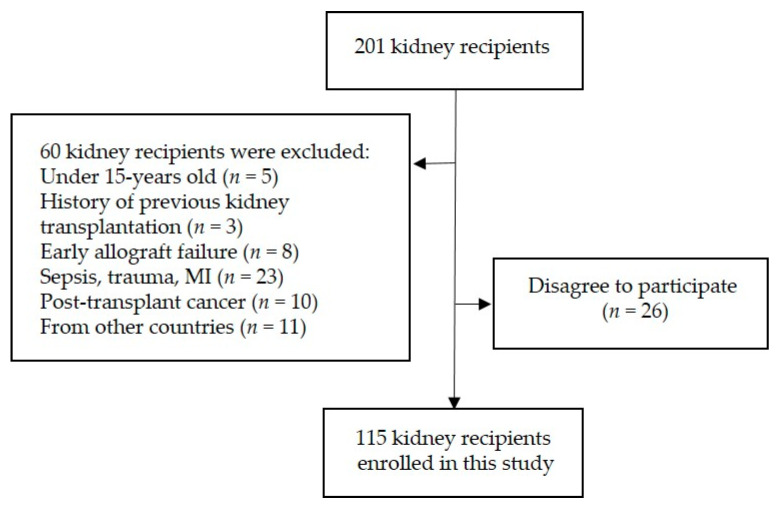
A flow-chart of patients selection.

Out of 200 renal recipients, 115 patients (72 males/43 females) were included (Figure 1). The demographic and clinical characteristics of the participants are presented in Table 2. Participants were examined in terms of VDR and VDBP polymorphisms. Most of the kidney recipients were treated by a triple immunosuppression therapy including corticosteroids (prednisolone), mycophenolate mofetil (MMF), and Tacrolimus that was tailored based on measured plasma levels.

### 3.1. Frequencies of VDR and VDBP Alleles and Genotypes Distribution

**Figure 2:**
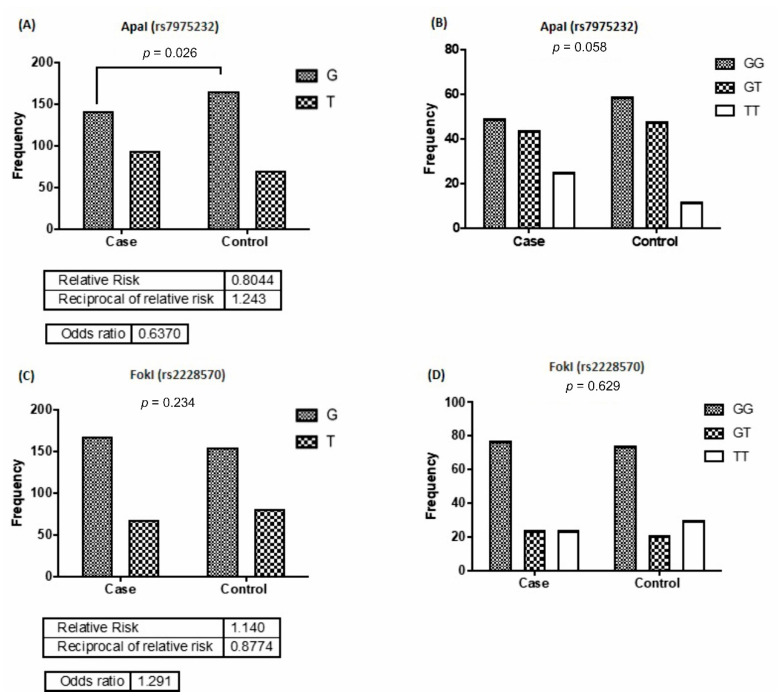
Allele and genotype frequencies of vitamin D receptor (VDR) polymorphisms in the studied groups. Comparing the *ApaI* (**A**,**B**) and *Fok*I (**C**,**D**) polymorphisms in kidney recipients and control groups.

**Figure 3:**
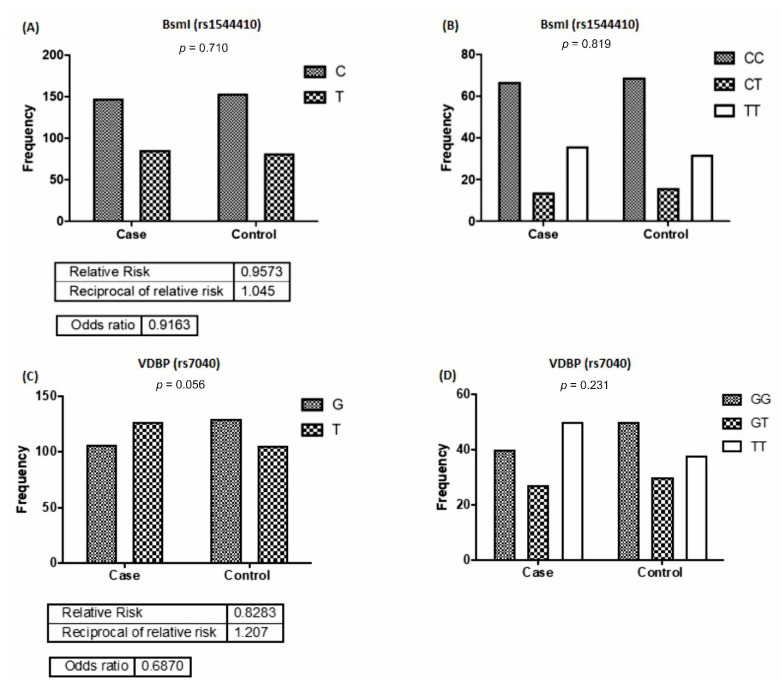
Allele and genotype frequencies of *Bsm*I and vitamin D-binding protein (VDBP) rs7040 polymorphisms in the studied groups. Comparing the *Bsm*I (**A**,**B**) and rs7040 (**C**,**D**) polymorphisms between kidney recipients and control groups.

The frequency of alleles and genotypes distribution in *Apa*I (rs7975232) are shown in Figure 2A,B. The results showed that the G allele frequency in rs7975232 polymorphism in the control group was significantly 0.63 times more than that of the case group (*p* = 0.026). There were differences between the two groups concerning the distribution of TT, GG, and GT genotypes of rs7975232; however, they were not statistically noteworthy (*p* = 0.058). Comparing the case and control group, concerning the frequency of *Fok*I (rs2228570) G and T alleles (*p* = 0.234), no significant difference was observed (Figure 2C). Furthermore, the frequency of rs2228570 genotypes (GG, GT, and TT) was not statistically significant between the groups (*p* = 0.629) (Figure 2D). Similarly, no significant differences were detected between the frequency of T and C alleles (*p* = 0.710) and that of CC, CT, and TT genotypes (*p* = 0.819) of the *Bsm*l genotype (rs1544410) between the two groups (Figure 3A,B). Furthermore, no significant difference was detected in the frequency of G and T alleles (*p* = 0.056) and that of the GG, GT, and TT genotypes of rs7040 (*p* = 0.230) between the case and control groups (Figure 3C,D).

### 3.2. VDR and VDBP Gene Polymorphisms Based on Allograft Rejection

**Figure 4:**
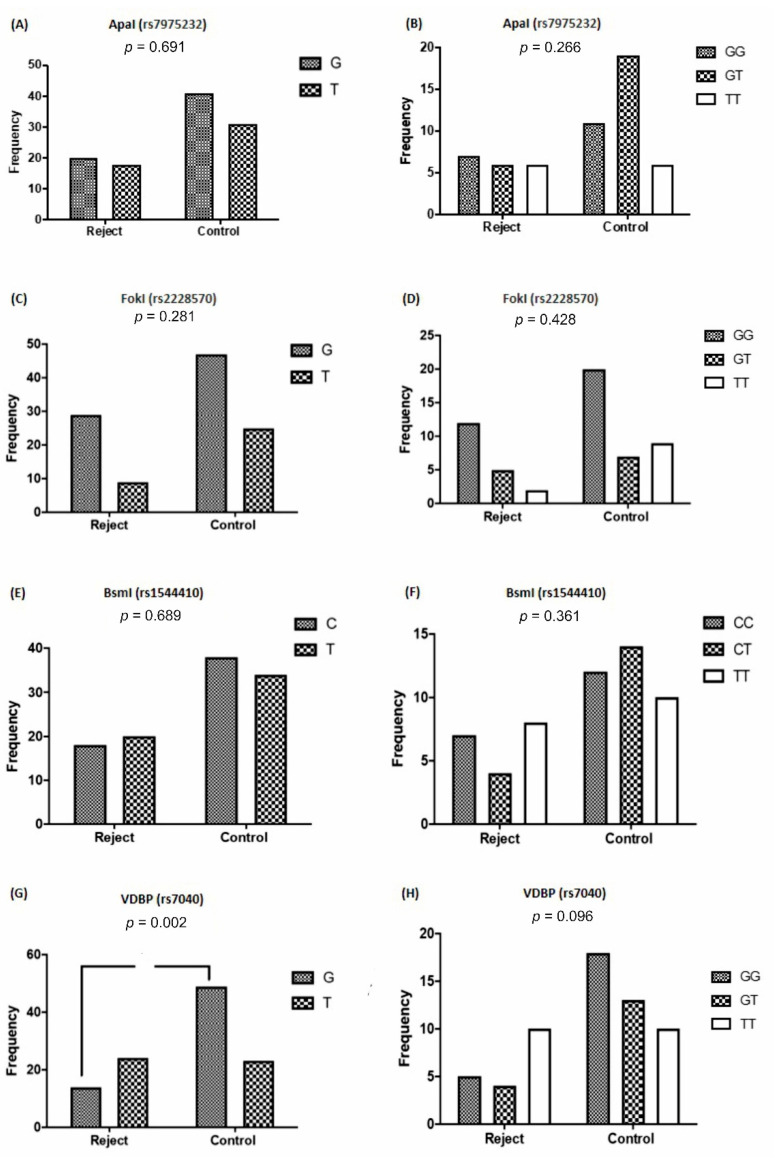
Frequencies of VDR and VDBP polymorphisms in the allograft rejection and healthy groups. (**A**,**B**) *Apa*I, (**C**,**D**) *Fok*I, (**E**,**F**) *Bsm*I, and (**G**,**H**) rs7040 polymorphisms. VDR: vitamin D receptor; VDBP: vitamin D-binding protein.

Out of the 115 cases of kidney transplants, 21.7% had a history of transplant rejection. The comparison of the frequency of VDR and VDBP alleles and genotypes distribution, concerning the graft rejection are depicted in Figure 4. As it can be seen, no significant difference was shown concerning the frequency of G and T alleles (*p* = 0.691) and the distribution of rs7975232 genotypes (*p* = 0.266), regarding the graft rejection (Figure 4A,B). Similarly, in the rs2228570 genotype, there was no significant difference in the frequency of G and T alleles (*p* = 0.281) and the distribution of the GG, GT, and TT genotypes (*p* = 0.428) between the acute rejection and the control groups (Figure 4C,D). Additionally, no significant change was detected between the frequency of T and C alleles (*p* = 0.689) and the distribution of the rs1544410 genotypes (CC, CT, and TT) (*p* = 0.361) (Figure 4E,F). As it can be seen in Figure 4G, the frequency of the G allele in the rs7040 genotypes was significantly low (*p* = 0.002) in patients with allograft rejection. Moreover, this allele increased the risk of transplant rejection to 0.27. The distributions of rs7040 GG and GT genotypes were lower in the rejection group; however, they were not statistically significant (*p* = 0.096) (Figure 4H).

### 3.3. The Frequency of VDR and VDBP Gene Polymorphisms Based on Viral Infection

**Figure 5:**
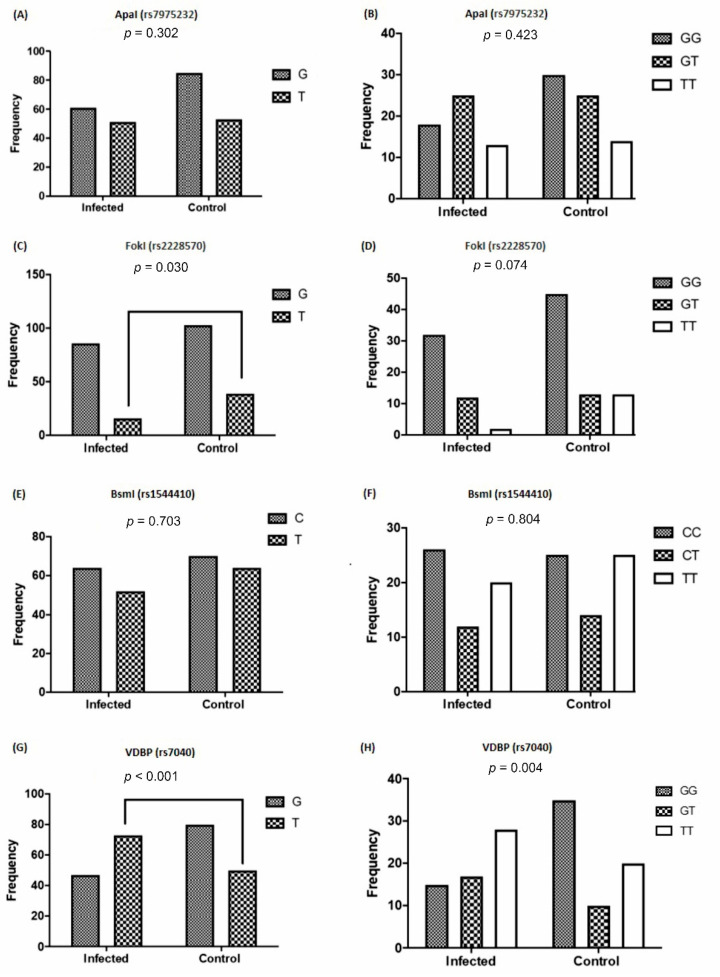
Comparison of the allele and genotype frequencies of VDR and VDBP polymorphisms in the recipients with a history of viral infection and healthy individuals. (**A**,**B**) *Apa*I, (**C**,**D**) *Fok*I, (**E**,**F**) *Bsm*I, and (**G**,**H**) rs7040 polymorphisms. VDR: vitamin D receptor; VDBP: vitamin D-binding protein.

The comparison of the frequency of the studied genotype alleles, regarding the history of viral infections is depicted in Figure 5. No significant differences were observed in the frequency of G and T alleles (*p* = 0.302) and the distribution of the *Apal* genotypes between the recipients with the history of viral infection and the controls (Figure 5A,B). In the rs2228570 genotype, the frequency of T allele in patients with a history of viral infection was significantly lower than the controls (*p* = 0.030). This T allele showed a risk history of viral infections (Odd ratio = 2.035) (Table 3). The distribution of the TT rs2228570 genotype was also lower in the recipients with a history of viral infection, however, it was not significant (*p* = 0.074) (Figure 5C,D). The frequency of C and T alleles (*p* = 0.703) and the distribution of rs1544410 genotypes (*p* = 0.804) displayed no significant difference between the control and viral infection groups (Figure 5E,F).

The T allele frequency of rs7040 was significantly higher in cases with viral infections (*p* < 0.001) compared with the controls, with an Odd ratio of 0.40 (Table 3). Moreover, the distribution of the GG genotype of rs7040 was significantly lower (*p* = 0.004) in cases with a history of viral kidney infection compared with the controls (Figure 4 G,H).

### 3.4. Connection of Vitamin D Levels with VDR and VDBP Gene Polymorphisms

**Figure 6:**
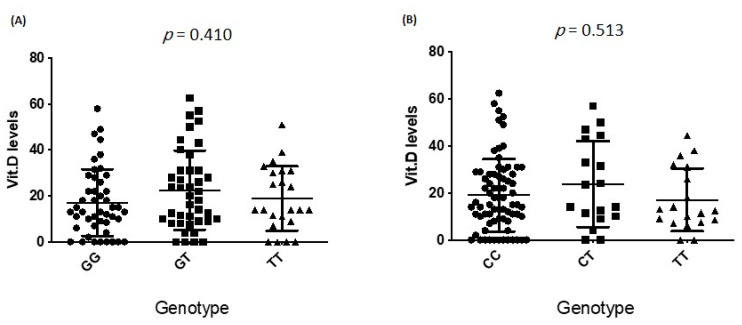
Association of vitamin D status with VDR and VDBP gene polymorphisms. Association of vitamin D status with (**A**) VDR and (**B**) VDBP genotypes. VDR: vitamin D receptor; VDBP: vitamin D-binding protein.

The comparison of various VDBP and VDR polymorphisms genotypes, pertaining to the amount of vitamin D in the patients under study indicated that there was no difference between the frequencies of various polymorphisms genotypes distribution related to vitamin D (*p* ≥ 0.410), Figure 6A,B.

## 4. Discussion

Our results showed that the VDR (*Fok*I) and VDBP (rs7040) alleles and their genotype distribution were significantly associated with graft outcome. The G allele frequency in VDBP rs7040 polymorphism was significantly lower in recipients with allograft rejection. In the *Apal* rs7975232 polymorphism, the frequency of the G allele in the kidney transplant recipients was lower than those of the healthy individuals. Significant variances were detected between the frequencies of the VDR *Fok*I and VDBP rs7040 alleles and viral infection in the studied groups. Moreover, the GG genotype distribution was low in recipients with a history of viral infection.

The recent discovery of VDR and its abundant binding sites have surged an interest to study its effect in different biological processes throughout the body [7]. The nutritional deficit, lower physical inactivity, low sunlight exposure, and elevated amounts of fibroblast growth factor 23 (FGF-23) can cause vitamin D deficiency. Additionally, decreased serum albumin, liver malfunction, diabetes, and corticosteroid therapy are attributed to decreased vitamin D levels post-transplantation [21]. Up to 97% of cases had vitamin D insufficiency and about 39% of patients exhibited vitamin D deficiency after organ transplantation [22]. Vitamin D insufficiency has been detected not only for a short period after kidney transplant, but also for a very long time in patients never supplemented with sterols [23]. In a cross-sectional investigation (*n* = 94 patients), renal graft recipients with lower levels of 25(OH)D exhibited a higher rate of acute rejection [24]. In another investigation, it was shown that patients with 25(OH)D levels less than 10 ng/mL were more exposed to acute rejections [14] proposing the potentially pivotal role of vitamin D in the preservation of graft function. A cut-off level of 15 ng/mL three months after transplantation was suggested to be an independent risk factor for GFR decline and tubular atrophy and fibrosis one year after transplantation [25]. Even though the exact mechanism of this association is unknown, the role of genetic variations of VDR and VDBP should be taken into account. Different polymorphisms have been recognized in the VDR gene on chromosome 12. Examples include RFLPs such as *Tru*9I, *Bsm*I, *Taq*I, *Apa*I, and *EcoRV* in exons 8 and 9 [18,26,27]. Moreover, a T to C variation in exon 2 generated another RFLP in the VDR gene termed *Fok*I [28]. It has been shown that two genetic variations in VDBP (rs7040, rs4588) can result in alterations in the sequence and function of this protein [6]. VDR polymorphisms are associated with adverse outcomes of transplantation such as the increased risk of graft rejection and viral infections [14,15], the severity of secondary hyperparathyroidism, bone density of kidney recipients [29], and an increased risk for bone diseases after transplantation [30]. It is also reported that the VDR *Fok*I polymorphism is a genetic risk factor for the development of post-transplantation diabetes mellitus [31].

Falleti et al. proved that the presence of G-*-T/G-*-T diplotypes of the *Taq*I T > C, *Apa*I T > G, and *Bsm*I G > A polymorphisms were connected with a high risk of acute cellular rejection and CMV infection in liver transplanted patients [32]. However, connection between *Bsm*I and *Fok*I polymorphisms and the incidence of acute rejection was not proved in another study [33]. Moreover, the association between kidney allograft survivals with VDR *Fok*I T allele has been reported; however, no significant association was found between acute rejection and *Apa*I and *Fok*I genotypes [34]. Similar to this result, in the present work, no statistically significant association was observed between the *Apa*l, *Bsm*I, and *Fok*I genotypes and acute rejection. In patients with the rs4588 VDBP polymorphisms, the GC genotype has been in relation with increased rate of graft survival in kidney transplantation [16]. In our study also, in VDBP rs7040 polymorphism, the G allele frequency significantly was lower in patients with transplant rejection with an Odd ratio of 0.27.

It has been shown that 1,25(OH)_2_D triggers the expression of TLR2 and cathelicidin in epithelial and myeloid cells [35]. Moreover, it is increased as a defense against M. tuberculosis infection [36]. In addition, 1,25(OH)_2_D prohibits the development of interferon-gamma and interleukin 2 (IL-2) generating Th1 and Th17 cells [37]. Several studies have explored the connection between certain viral infections such as the Epstein–Barr virus (EBV), human immunodeficiency virus (HIV), hepatitis B and C, and influenza and vitamin D levels [38]. However, the result of a study showed that vitamin D deficiency independently increases the rejection risk, but does not escalate the risk of BK and CMV infection in 90 days monitoring post-transplantation [12,15].

In our study, significantly high frequency of the T allele and low distribution of GG genotype in VDBP rs7040 genotype were seen in recipients with a history of viral infection. Moreover, low frequency of the VDR *Fok*I T allele was observed in this group compared with the controls. Our Logistic regression analysis showed that the VDR *Fok*I and VDBP rs7040 polymorphisms could significantly increase the Odds ratio of viral infection. 

The main limitation of this study was the lack of the biologically active form of Vitamin D (1,25(OH)_2_ D) measurement that could more identify the pathogenic link between post-transplant outcome (viral infection and graft rejection) and vitamin D status. Small sample size was another limitation of this study that may affect the strength of the findings.

## 5. Conclusions

Overall, the results imply that (i) a low frequency of the G allele of the *Apa*I polymorphism exists in kidney transplant cases, (ii) a low frequency of the G allele of the rs7040 polymorphism is associated with poor graft function or rejection, (iii) a low frequency of the T allele of the *Fok*I polymorphism and a high frequency of the T allele and a low GG genotype distribution in the rs7040 VDBP polymorphism are associated with viral infections in kidney recipients. VDR *Fok*I and VDBP rs7040 polymorphisms may be the genetic factors correlated with renal allograft outcomes in our population.

## Figures and Tables

**Table 1:** Primer sequences.

Polymorphisms	Position	Description	Sequences (5′-3′)
rs2228570	chr12:47879112 (GRCh38.p12)	NG_008731.1:g.30920T > G; NG_008731.1:g.30920T > C; NG_008731.1:g.30920T > A	F: 5′-GTGGGTGGCACCAAGGAT-3′ R: GTCTCCACACACCCCACAGAT-3′
rs1544410	chr12:47846052 (GRCh38.p12)	NG_008731.1:g.63980G > T; NG_008731.1:g.63980G > C; NG_008731.1:g.63980G > A	F: 5′-CTGGGGCCACAGACAGG-3′ R: CCTGCCCGCAAGAAACCTCAA-3′
rs795232	chr12:47845054 (GRCh38.p12)	NG_008731.1:g.64978G > T	F: 5′-GGCAGTGGTATCACCGGTCAG-3′ R: 5′-CTGTGGGCACGGGGATAGAGA-3′
rs7040			F: 5′-TTGCCTGATGCCACACCC-3′ R: 5′-GGAACAGCAGTTGGAGGCAAA-3′

**Table 2:** Demographic and baseline clinical data.

Variables	Controls	Cases	*p*-Value
No. of cases	100	115	-
Demographic characteristics			
Age mean ± SD (years)	41.3 ± 3.2	41.8 ± 5.3	0.760
Gender, Male, *n* (%)	58 (58%)	72(62.6%)	0.810 ^a^
Female, *n* (%)	32 (32%)	43(37.4%)	
BMI, Kg/m^2^ (mean ± SD)	20.92 ± 4.70	22.43 ± 3.30	*p* = 0.125
Smoking history, *n* (%)	25(10)	15(7)	*p* = 0.092 ^a^
Clinical characteristics			
Urea (mg/dL)	27.46 ± 1.7	58.02 ± 23.7	<0.001
Serum creatinine (mg/dL)	1.04 ± 0.1	1.53 (1.15)	<0.001 ^b^
Serum Calcium (mg/dL)	8.99 ± 0.75	7.16 ± 1.27	*p* = 0.041
Phosphate (mg/dL)	3.8 ± 0.42	4.61 ± 0.81	*p* = 0.030
Serum 25(OH) D (ng/mL)	40.02 (14.38)	16.50 (20.38)	*p* < 0.001 ^b^
GFR (mL/min/1.73 m^2^)	84.65 (20.12)	59.89 (31.32)	<0.001 ^b^
PTH (pg/mL)	43 (15)	141 (30)	*p* = 0.024 ^b^
Hemoglobin ^c^	14.71 ± 1.84	11.42 ± 5.23	*p* = 0.051
Albumin (g/dL) ^d^	4.31 ± 0.95	3.24 ± 1.03	*p* = 0.062
Pre-transplant dialysis (months)	-	25.60 ± 10.45	
Time post-transplantation (months)	-	65 (40.21)	
Underlying ESRD, *n* (%)			
Glomerulonephritis	-	48 (41.73)	
Interstitial nephropathy	-	31 (26.95)	
Diabetic	-	14 (12.17)	
Hypertension	-	9 (7.82)	
Unknown	-	5 (4.37)	
Polycystic kidney disease	-	3 (2.60)	
Vasculitis	-	3 (2.60)	
Reflux nephropathy	-	2 (1.73)	
Post-transplant complications, *n* (%)			
DGF	-	5 (4.34)	
Acute allograft rejection	-	25 (21.70)	
Chronic graft dysfunction ^e^	-	23(20)	
New-onset diabetes	-	9 (7.8)	
CMV, *n* (%)	-	20 (17.4)	
BK polyomavirus, *n* (%)	-	16 (13.9)	
CMV + BK polyomavirus, *n* (%)	-	19 (16.5)	
CMV + Parvovirus	-	1 (0.87)	
Chronic allograft failure ^f^	-	15 (13.04)	
Recipients survival	-	115 (100)	

eGFR: estimated glomerular filtration rate; ESRD: end-stage renal disease; DGF: Delayed graft function, 25(OH)D: 25-hydroxyvitamin D. Numbers are presented as mean ± SD. ^a^ Number (%) is presented. *p*-value is based on Chi-squared test. ^b^ Median (Interquartile Range) is presented and *p*-value is based on Mann–Whitney U test. ^c^ Data on Hemoglobin did not available for 5 recipients. ^d^ Data on Albumin did not available for 11 recipients. ^e^ Chronic graft dysfunction is defined as Cr > 3 and GFR ≤ 22.9. ^f^ Chronic allograft failure is considered as Cr > 5, GFR ≤ 10, and a need for hemodialysis.

**Table 3:** The correlation between the genotypes and the allograft outcome.

Risk Factors	OR (95%CI)	*p* Value
rs1544410 and graft rejection	0.8053	0.6892
rs2228570 and graft survival	1.714	0.2817
rs7975232 and graft survival	0.8401	0.6910
rs7040 and graft survival	0.2738	**0.0023**
rs1544410 and viral infection	1.125	0.7032
rs2228570 and viral infection	2.035	**0.0309**
rs7975232 and viral infection	0.7458	0.3021
rs7040 and viral infection	0.4024	**0.0006**

OR: odds ratio. *p* values < 0.05 are shown in bold.

## Data Availability

The data presented in this study are available on request from the corresponding author.
